# Medical Malpractice Lawsuits Involving Percutaneous Coronary and Peripheral Intervention

**DOI:** 10.1016/j.jscai.2023.100975

**Published:** 2023-04-21

**Authors:** Gabrielle Kirshteyn, Roei Golan, Ian C. Gilchrist, Mauricio G. Cohen

**Affiliations:** a Florida State University College of Medicine, Tallahassee, Florida; b Cardiology Division, Department of Medicine, Pennsylvania State Heart and Vascular Institute, Hershey, Pennsylvania; c Heart and Vascular Institute, Cleveland Clinic Florida, Weston, Florida

**Keywords:** malpractice, medical malpractice, percutaneous coronary intervention, percutaneous peripheral intervention

## Abstract

**Background:**

Malpractice lawsuits following percutaneous coronary and peripheral interventions are not uncommon. Understanding the most common causes of medical legal action can inform clinicians and prevent future injury and litigation.

**Methods:**

LexisNexis is a legal database that compiles all publicly available court records for state and federal jury verdicts and settlements in the United States. We analyzed LexisNexis for malpractice cases involving percutaneous coronary and peripheral interventions between January 1, 2005 and December 31, 2020.

**Results:**

We found a total of 89 cases over the studied period, of which 55 cases were coronary interventions, 18 cases were peripheral interventions, and 16 cases did not specify the vessel location. Procedural error was alleged in 44 (49.4%) cases, complications in 42 (47.2%) cases, failure to monitor in 22 (24.7%) cases, failure to properly treat in 15 (16.9%) cases, incorrect treatment in 15 (16.9%) cases, failure to refer in 10 (11.2%) cases, lack of informed consent in 9 (10.1%) cases, and delay of intervention in 3 (3.4%) cases. The most common procedural error was vessel perforation (34.1%). Death was the most prevalent precipitating medical outcome of the cases (n = 41, 46.1%). Litigation resolution favored the defendant in 43 (48.3%) cases, the plaintiff in 13 (14.6%) cases, and a settlement was reached in 33 (37.1%) cases.

**Conclusions:**

Awareness of the most common causes of litigations among interventional cardiologists and vascular specialists may prevent future legal actions and promote implementations of processes to improve patient care. Our results suggest the importance of attentive patient care before, during, and after percutaneous coronary and peripheral interventions. Procedural expectations and risks should be meticulously discussed with patients and families.

## Highlights

• 89 malpractice cases involving percutaneous coronary intervention and peripheral interventions were analyzed.
• Procedural errors and complications were the most common allegations.
• Vessel perforation was the most frequent procedural error.
• Death was the most prevalent precipitating medical outcome.
• Findings underscore the importance of attentive patient care and informed consent.

## Central Illustration

**Figure:**
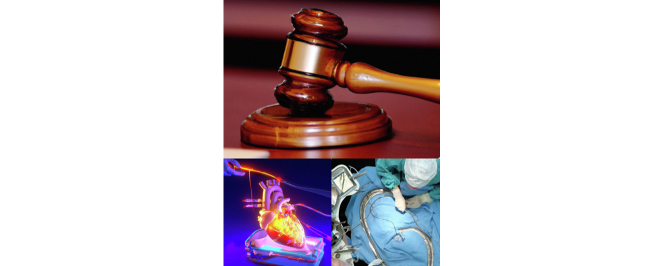

## Introduction

Percutaneous revascularization procedures are minimally invasive interventions widely used to restore blood flow in different vascular territories.^1^ Even though they are safe and effective, they are not without complications. In fact, these complications can lead to malpractice litigations, which are not infrequent (Central Illustration). In a span of 22 years (1992 to 2014), there was a total of 280,368 paid malpractice claims involving 175,667 physicians of varying specialties.^2^ Specifically, it was found that 18.5% of 871 vascular surgeons were named in a malpractice case within the last 2 years.^3^

**Central Illustration:**
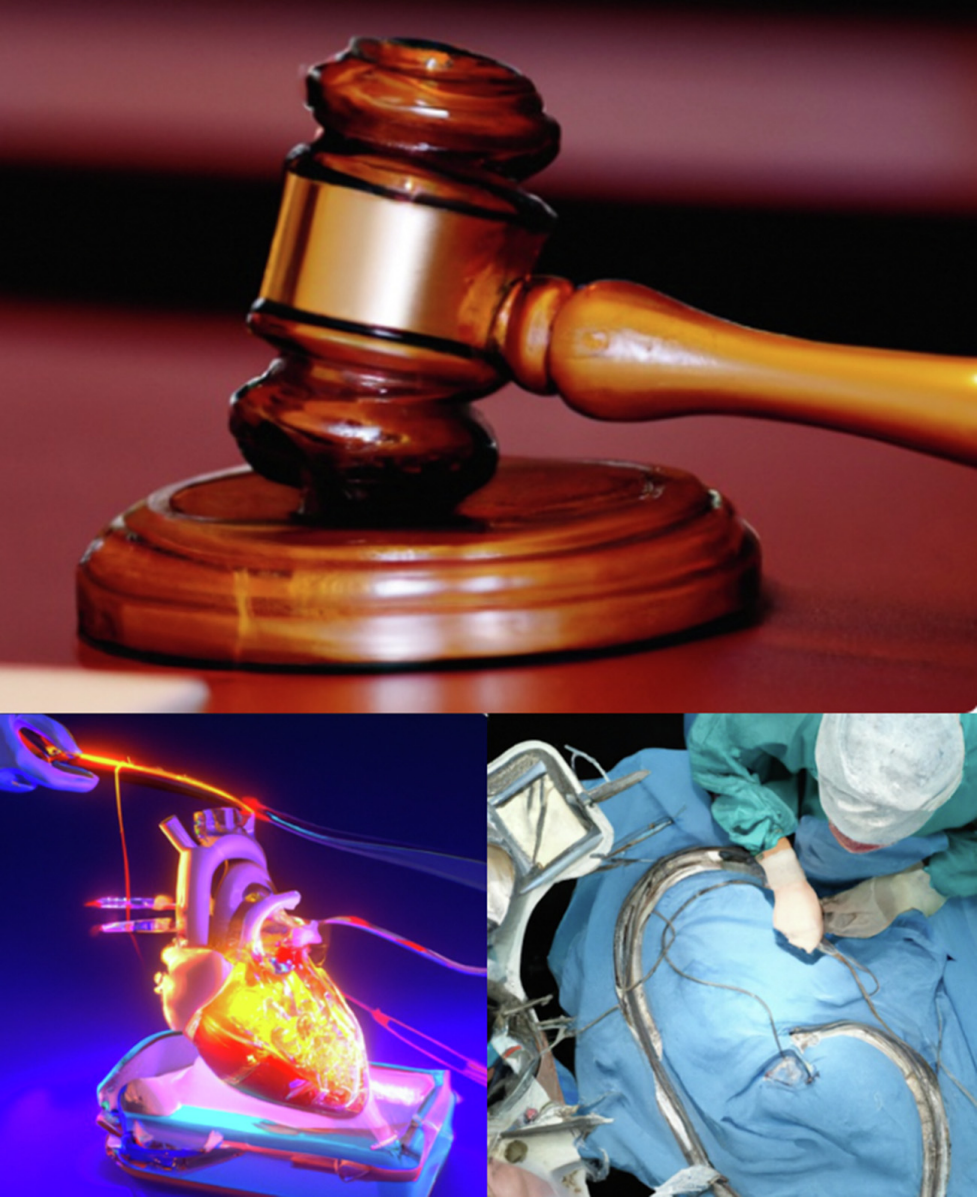
It is not unusual for malpractice lawsuits to arise after percutaneous coronary and peripheral interventions. Clinicians can prevent future injury and litigation by understanding the most common reasons for medical legal action.

Analyzing malpractice cases could elucidate the shortcomings of revascularization procedures and inform both patients and physicians. Malpractice education can help physicians anticipate complications, recognize how to minimize mistakes, and be better equipped to deal with errors. There is a paucity of information on the characteristics of malpractice claims involving percutaneous revascularization procedures. We aimed to collect and analyze malpractice claims involving coronary and peripheral revascularization procedures and investigate their trends.

## Methods

Medical malpractice litigations regarding percutaneous revascularization procedures were collected through the LexisNexis Academic legal database. LexisNexis compiles all publicly available court records for state and federal jury verdicts and settlements in the United States and is a leading tool for medical malpractice studies.^4^ The search criteria for this investigation included medical malpractice cases involving percutaneous coronary and peripheral interventions between January 1, 2005, and December 31, 2020. To identify relevant cases, we searched in a Boolean manner using the keywords: [(angioplasty or “cardiovascular angioplasty” or “coronary angioplasty” or “balloon angioplasty” or “percutaneous coronary intervention” or “laser angioplasty” or “angioplasty with stent” or “vascular angioplasty”) and (malpractice)]. All resulting cases were manually reviewed to include only those that were relevant to the study. Data was individually extracted from each litigation. Cases were excluded if the patient did not receive a percutaneous coronary or peripheral intervention.

The recorded data included patient age, vessel location, state, verdict type, award size, precipitating medical outcome, and the plaintiff’s alleged reasons for litigation. The alleged reasons for litigation were then further divided into 8 categories:

1. Delay in treatment: revascularization procedure, associated medications, or laboratory tests were not performed in a reasonable time frame and resulted in injury. For example, a delayed percutaneous coronary intervention resulted in the patient experiencing a myocardial infarction.
2. Failure to monitor: failure to adequately monitor a patient’s condition resulted in a complication. For example, retroperitoneal bleeding after femoral artery access was not noted because vital signs were ignored.
3. Procedural error: an error of execution or planning of a procedure that resulted in an injury. For example, the wrong size balloon was used, resulting in a vessel perforation.
4. Lack of informed consent: neglecting to provide patient appropriate information about the risks, benefits, and alternatives to a surgery or treatment, and/or obtaining the patient’s permission to undergo the procedure.
5. Failure to properly treat: the physician did not fully and properly address or treat the condition with the revascularization procedure. For example, an occluded coronary vessel was not recognized at the time of catheterization, resulting in an acute myocardial infarction not being reperfused.
6. Complication: a direct, undesirable result of the procedure or treatment.
7. Failure to refer: the physician failed to refer the patient to another clinician or specialist and this act resulted in injury. For example, a physician did not refer to a physician with greater experience when the patient needed a procedure the physician was not skilled to perform.
8. Incorrect treatment: the physician made the correct diagnosis but performed the wrong treatment. For example, a patient received coronary stenting instead of indicated coronary artery bypass surgery.

The listed categories are not mutually exclusive, as one case could have multiple allegations.

Categorical data are presented as frequencies with their respective percentages. Continuous data are presented as means with range. Our analysis is purely descriptive, and no statistical comparisons were made.

## Results

**Figure 1:**
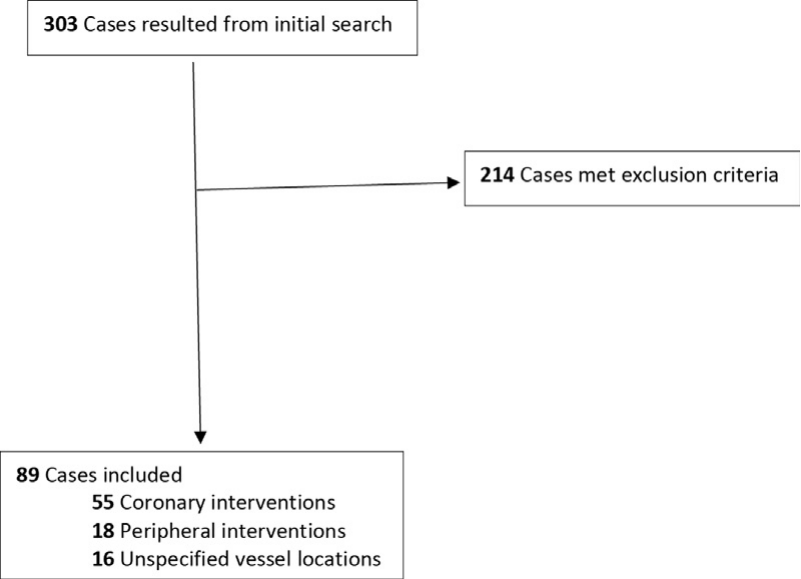
**Flow diagram of inclusion criteria**.

The LexisNexis database search yielded a total of 303 litigations (Figure 1). After a thorough manual review, 89 cases fit the inclusion criteria and were the subject of data analysis. The 89 lawsuits that were included involved 55 coronary interventions, 18 peripheral interventions, and 16 that did not specify the vessel location.

**Figure 2:**
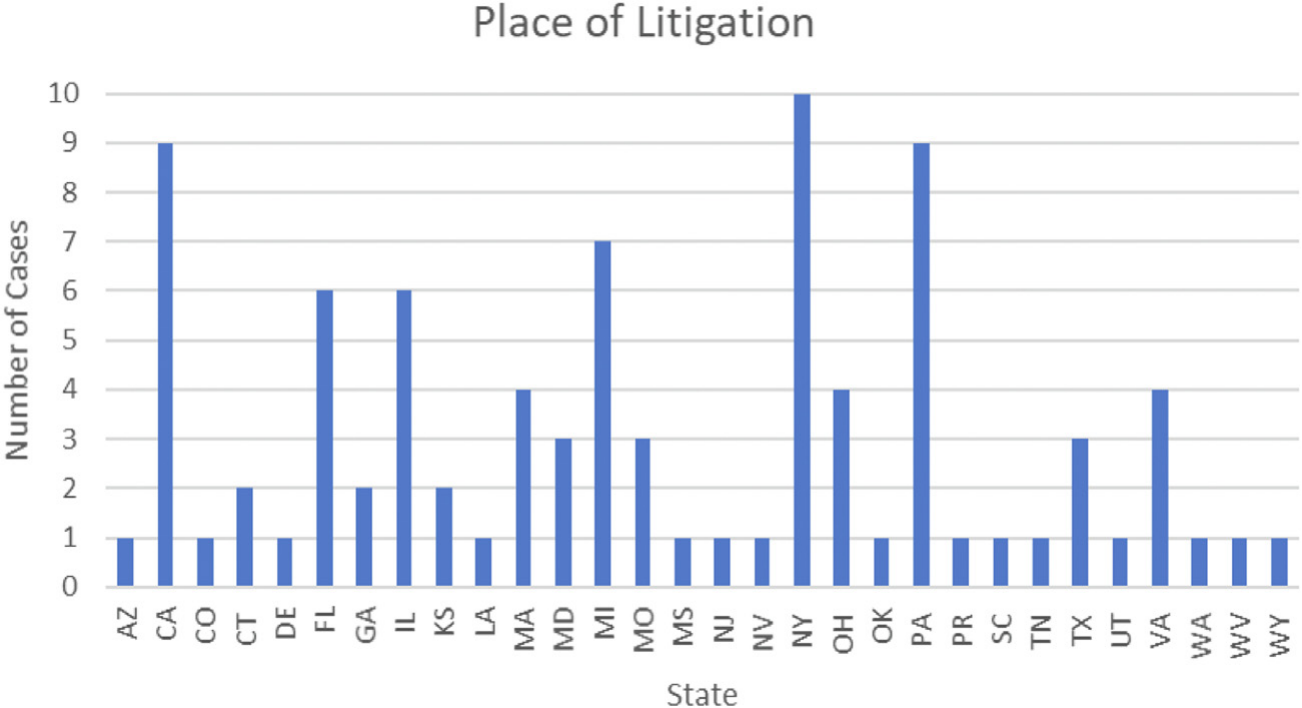
**Litigations by state**.

Among the malpractice cases analyzed, 42 cases (47.2%) included the patient’s age (median 61 years, range 2-88). The litigations took place in 30 states, with New York (n = 10, 11.2%) having the most, followed by California (n = 9, 10.1%) and Pennsylvania (n = 9, 10.1%) (Figure 2).

In terms of litigation resolution, 43 cases (48.3%) resulted in the jury rendering the defendant (the physician) not liable. There were 13 (14.6%) verdicts that favored the plaintiff and 33 (37.1%) settlements. The average award amount of plaintiff verdicts was $5,189,613 (n = 13), ranging from $160,504 to $17,000,000. Of the settlements, 23 cases had undisclosed award amounts. The 10 cases that disclosed the settlement had a mean award amount of $1,934,000, ranging from $50,000 to $3,500,000.

**Figure 3:**
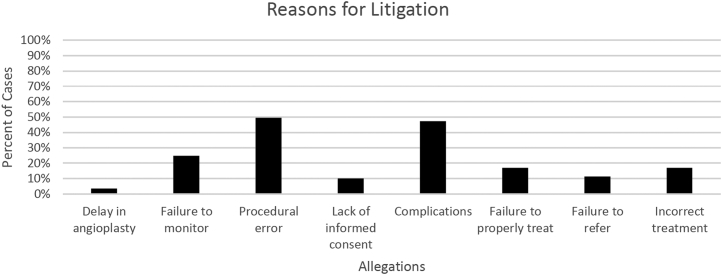
**Reasons for litigation based on plaintiff allegations**.

Procedural error was alleged in 44 (49.4%) cases, complications in 42 (47.2%) cases, failure to monitor in 22 (24.7%) cases, failure to properly treat in 15 (16.9%) cases, incorrect treatment in 15 (16.9%) cases, failure to refer in 10 (11.2%) cases, lack of informed consent in 9 (10.1%) cases, and a delay in procedure in 3 (3.4%) cases (Figure 3).

**Figure 4:**
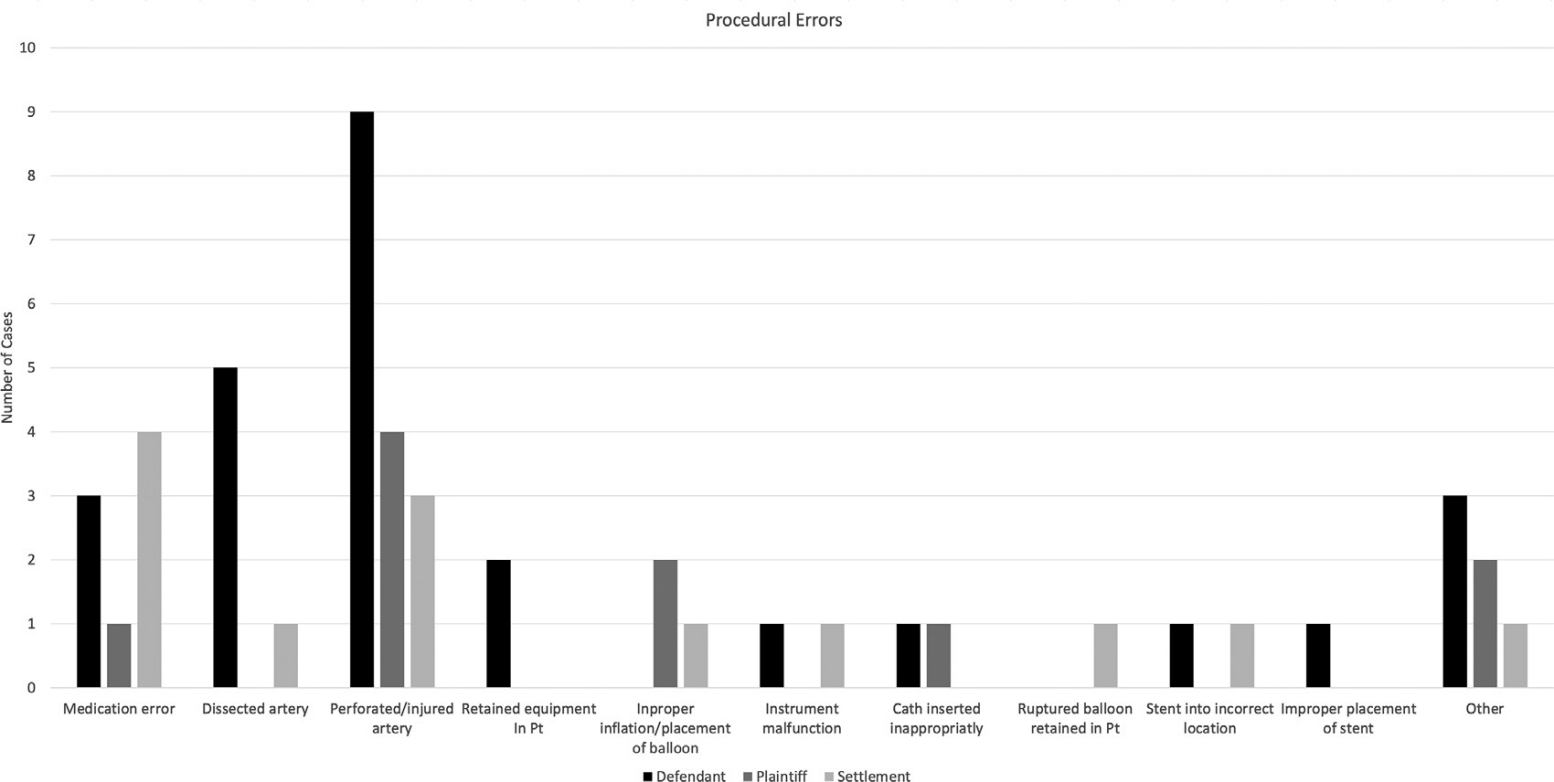
**Procedural errors made by physicians**.

The most common procedural error was vessel perforation (n = 15, 34.1%). Of the alleged procedural errors, 24 (53.3%) resulted in a defense verdict, 8 (17.8%) resulted in a plaintiff verdict, and 13 (28.9%) resulted in a settlement (Figure 4).

**Figure 5:**
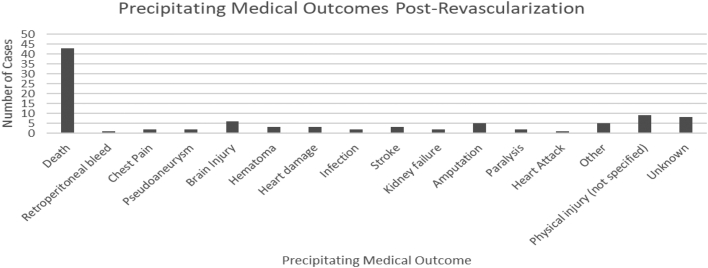
**Precipitating medical outcomes after revascularization.** Death following coronary and peripheral intervention was the most common outcome associated with the lawsuits.

**Figure 6:**
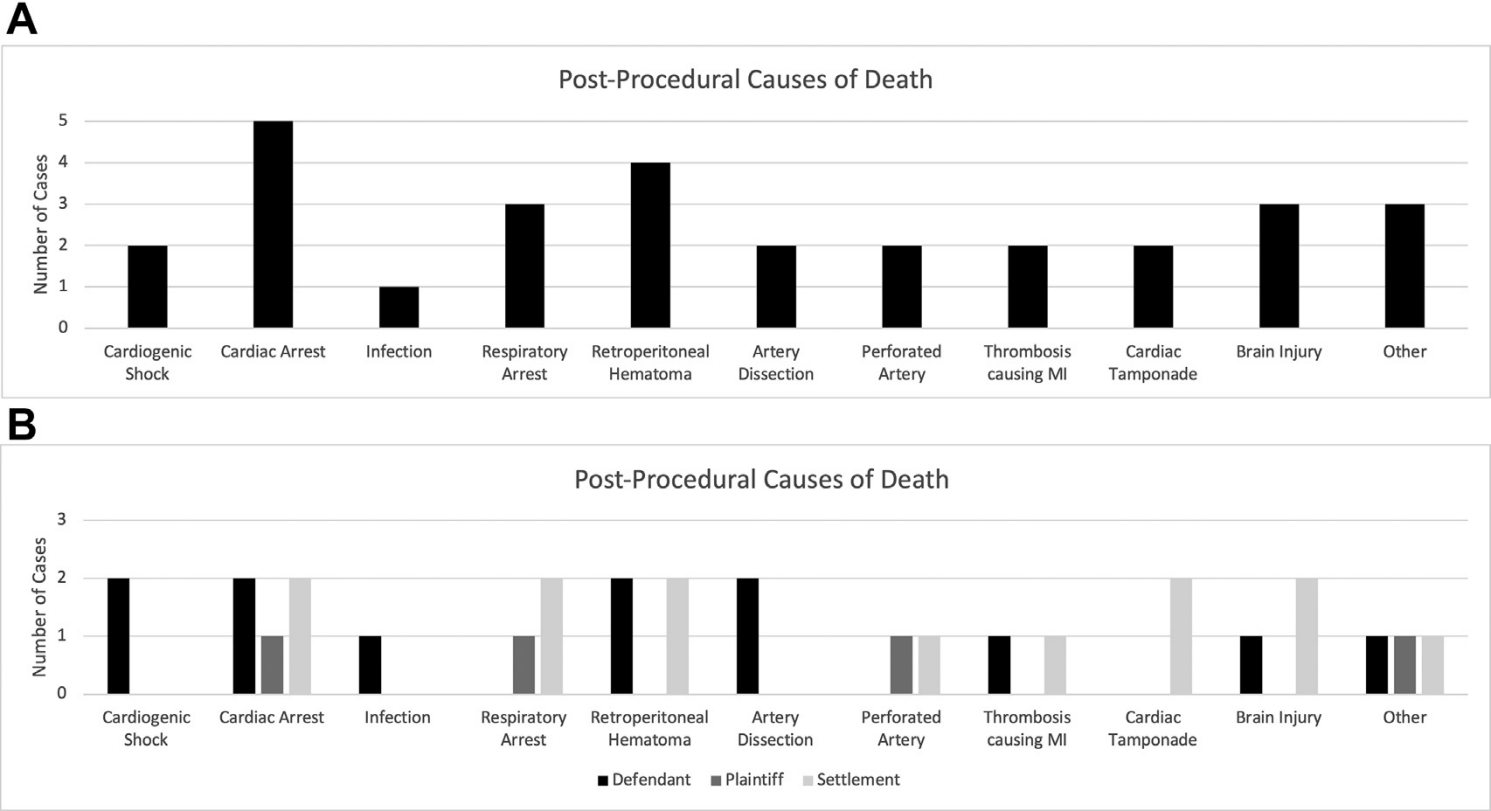
(**A**) **Illustrates postprocedural causes of death, and (B) breaks down whether the case resulted in verdicts for the defendants, plaintiffs, or in a settlement**.

Death was the most common adverse outcome precipitating malpractice cases (n = 41, 46.1%) (Figure 5). Of the cases that resulted in death, 28 stated the cause of death. Cardiac arrest was the most common cause (17.9%). Of the cases that led to death, 19 resulted in a defendant’s verdict (44.2%), 6 resulted in a plaintiff’s verdict (14.0%), and 18 resulted in a settlement (41.9%) (Figure 6).

## Discussion

In this study, we analyzed 89 medical malpractice allegations involving percutaneous coronary and peripheral interventions over the last 15 years to identify prevalent patterns that led to litigation. In the cases examined, almost 50% resulted in the jury rendering the defendant physician not liable, and a third resulted in settlement. The payment, in cases that favored the plaintiff, was significant and averaged $3,774,129, although the lack of transparency as to actual payments in many cases leaves the true damage assessment uncertain.

In 2018 alone, an estimated 480,000 percutaneous coronary interventions were performed in the United States.^5^ With advances in technology, percutaneous revascularization procedures have become safer, with low incidence of complications and perhaps an expectation of success by the patients. When complications do occur, it can lead to malpractice litigation, whether it was due to a true medical error or misplaced patient expectation.

We identified that the most common allegation against physicians was procedural error with vessel injury and perforation as the major cause. Our results were consistent with findings by Kim and Vidovich^6^ as they cited improper performance to be the most prevalent error involving coronary angiography. Similarly, Soh et al^3^ determined procedural error to be the most common malpractice allegation among vascular surgeons in malpractice litigation. Riley et al^7^ determined that the most common adverse event of coronary intervention was vessel perforation. In addition, we identified that death following coronary and peripheral intervention was the most common outcome associated with the lawsuits. Comparable results were found in the analyses by Kim and Vidovich^6^ and Soh et al^3^ as they concluded death to be the most prevalent postoperative injury (36% and 32%, respectively) reported in malpractice lawsuits involving percutaneous coronary interventions and vascular surgeons. Despite death being the most common outcome associated with the analyzed malpractice claims, percutaneous revascularization procedures are relatively safe as their median hospital risk-standardized mortality rate is 1.9%.^8^ The perception of safety in the general public may further enhance the risk of lawsuit when death occurs due to the unexpected nature of the outcome and unrealized expectations.

We found that most malpractice litigations involving percutaneous coronary and peripheral interventions resulted in defendant verdicts (48.3%). In contrast, two-thirds of malpractice cases among vascular surgeons had a defense verdict. Our calculated median plaintiff award was $4,086,004. This is substantially higher than the median plaintiff award of $1,830,000 calculated for vascular surgery litigations by Soh et al.^3^ Again, the lack of transparency to the awards in many cases may impair the validity of these values.

Lack of informed consent constituted 9.9% of malpractice litigations involving percutaneous coronary and peripheral interventions in our study. This issue has also been reported for other procedures such as coronary artery bypass grafting.^9^^,^^10^ Extensive and clear communication among the patient, the family, and the physician should occur before every procedure. Preventing inappropriate expectations and ensuring that the risks and benefits of the procedure are well understood are both crucial. If informed consent is not obtained, malpractice has occurred regardless of the procedural outcome. This highlights the foundational importance of proper consent.

Our study adds to the current body of evidence by providing a thorough overview of past physician errors that can be useful to recognize trends and problem areas in revascularization procedures. The current literature lacks a comprehensive analysis of medical malpractice allegations involving percutaneous coronary and peripheral interventions.

Our study is not without limitations. Finding reliable information on the outcomes of medico-legal actions in the United States is difficult. Much effort is placed in executing malpractice cases in the United States, but unfortunately, little information is ultimately made public in the end to help prevent further misfortunes. Settlements are sealed from public disclosure and results of jury awards are left unstated. Opportunities for learning from other misfortunes are limited.

The LexisNexis database used in our analysis only reports state and federal cases. Litigation through other mechanisms, such as out-of-court settlements, was not included in this study. Therefore, our sample does not encompass every medical malpractice litigation involving percutaneous coronary and peripheral interventions. Second, the cases provided include varying levels of detail; some cases reported enough details to address all of the categories analyzed in this study whereas others only provided enough information for a few variables. To confront this, we broadly searched LexisNexis and meticulously reviewed each case to incorporate as many relevant cases and datapoints as possible. Nonetheless, the information provided in this analysis serves as an educational tool to possibly prevent malpractice in the future.

Follow-up studies should investigate other legal databases or use multiple databases to compile litigation data. Additionally, future studies can broaden their inclusion criteria to include cases in which plaintiffs should have received revascularization interventions but did not, leading to alleged delay of treatment, improper care, and failure to treat coronary or peripheral vascular disease. Understanding the foundation of malpractice litigations involving percutaneous coronary and peripheral interventions could offer an opportunity for insight and improvement among physicians. The results reported in this study highlight the importance of meticulous perioperative conduct, adequate postoperative monitoring, and open communication between the physician and the patient and his/her family. By illuminating the most common sources of error, physicians and surgeons can become more vigilant to mistakes made in the past.
